# Supplementary material for: Structure of human NaV1.6 channel reveals Na+ selectivity and pore blockade by 4,9-anhydro-tetrodotoxin
Source: Nat Commun. 2023 Feb 23;14:1030. doi: 10.1038/s41467-023-36766-9 (PMC9950489; doi:10.1038/s41467-023-36766-9)
Supplement: Supplementary file 1 — Supplementary Information [file 41467_2023_36766_MOESM1_ESM.pdf]

Supplementary Information for

Structure of human $Na_V1.6$ channel reveals Na^+ selectivity and pore blockade by 4,9-anhydro-tetrodotoxin

Authors

Yue Li, Tian Yuan, Bo Huang, Feng Zhou, Chao Peng, Xiaojing Li, Yunlong Qiu, Bei Yang, Yan Zhao, Zhuo Huang, Daohua Jiang

This file contains Supplementary Figure 1-10 and Table 1-3.

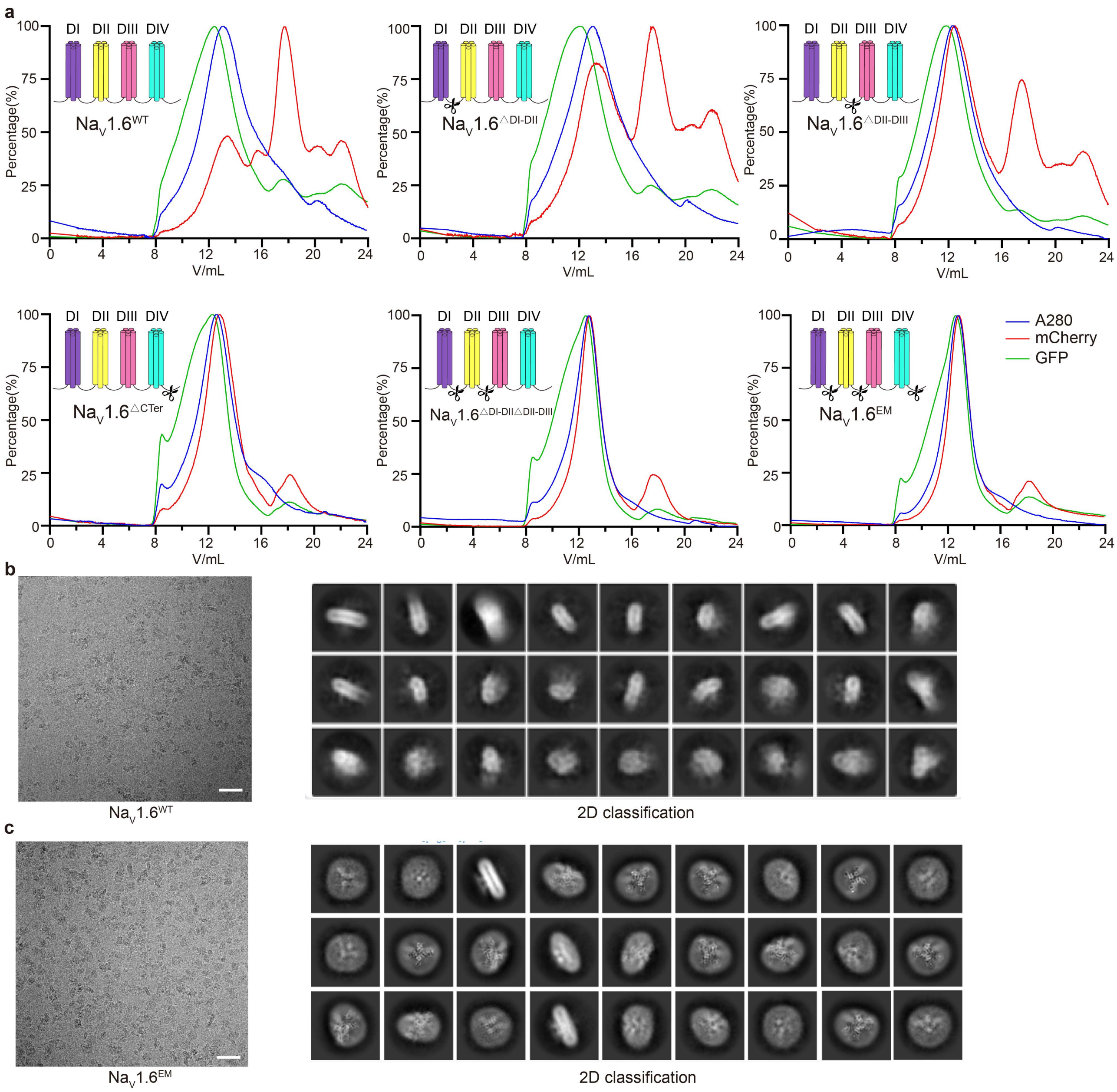

Supplementary Figure 1. Construct optimization of human $Na_V1.6/\beta1/\beta2$

a. Fluoresent SEC profiles of the $Na_V1.6$ WT and variants. The blue, red and green peaks represent singals from total protein absorbance at 280 nm, mCherry fluorecence and GFP fluoresence, respectively. Scissors indicate the truncation positions. **b-c**. Representative cryo-EM micrographs (Bar = 400 Å) and selected 2D class averages for $Na_V1.6^{WT}$ (**b**) and $Na_V1.6^{EM}$ (**c**). The experiments were repeated independently for more than 3 times with similar results.

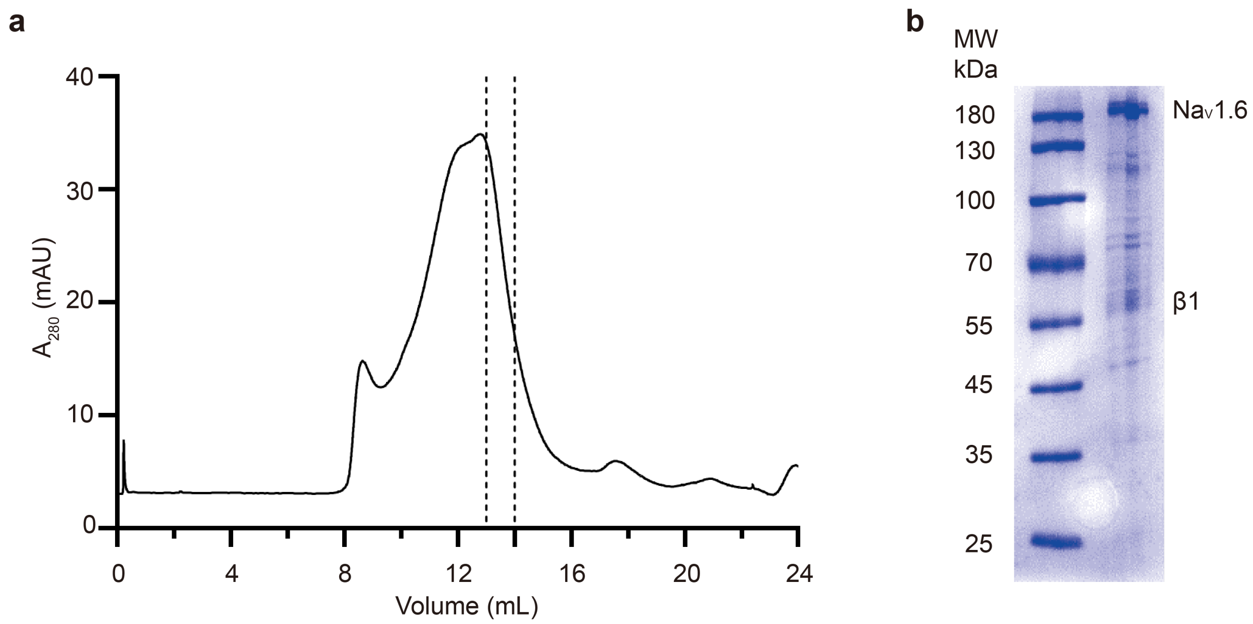

Supplementary Figure 2. Purification of human $Na_V1.6^{EM}$/β1/β2 complex

a. SEC profile of human $Na_V1.6^{EM}$/β1/β2 complex. Fractions between two black dashed lines were pooled and concentrated for cryo-EM analysis. **b**. SDS-PAGE gel of the purified $Na_V1.6^{EM}$/β1/β2 sample stained by Coomassie blue. Bands for $Na_V1.6^{EM}$ and β1 were labelled. The experiments were repeated independently for more than 3 times with similar results. Source data are provided as a Source Data file.

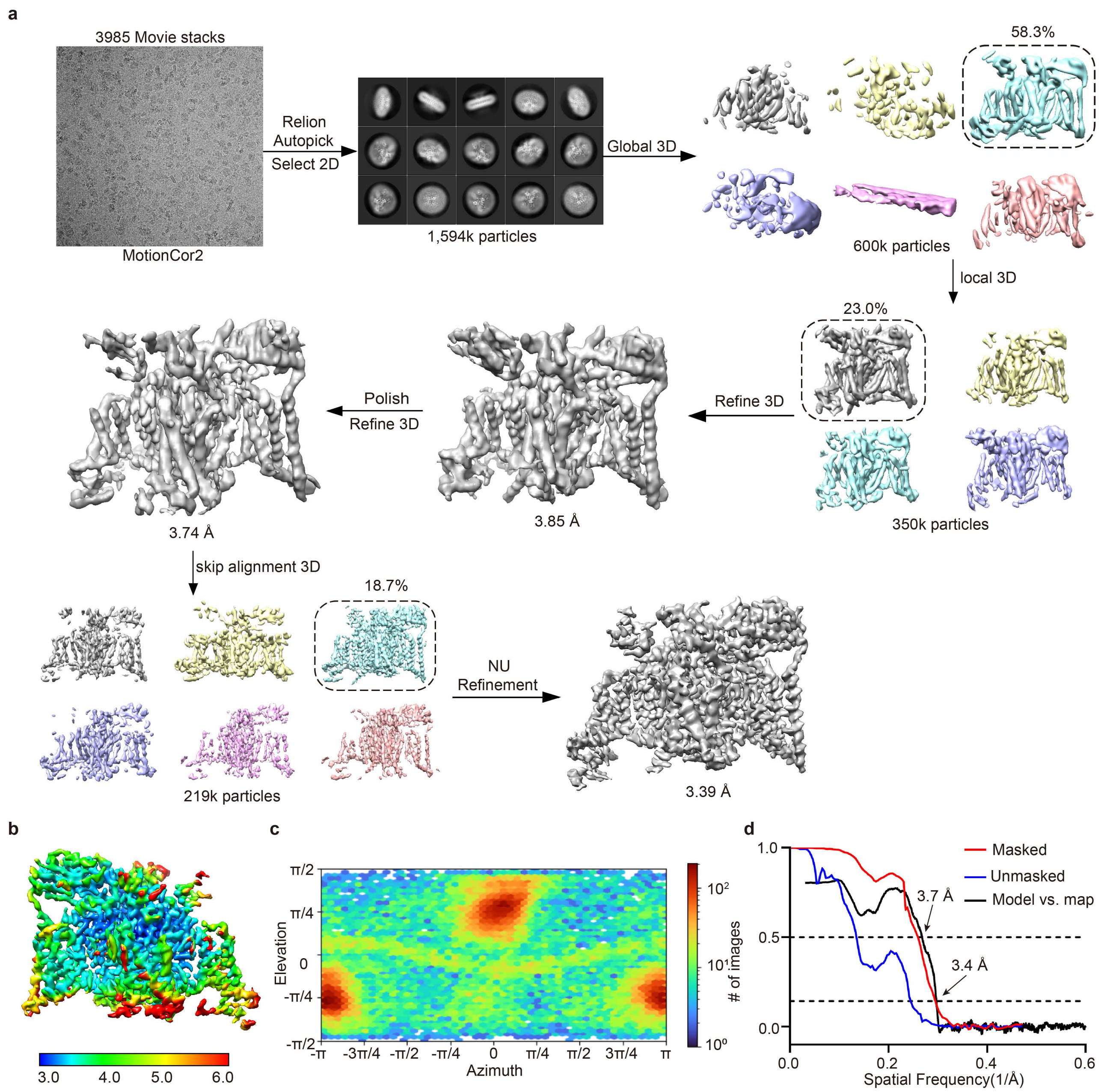

Supplementary Figure 3. Cryo-EM data processing of human $Na_V1.6^{EM}$/β1/β2 complex

a. The flowchart of cryo-EM data processing for $Na_V1.6^{EM}$/β1/β2 complex. A total of 1,594k particles were picked from 3,985 micrographs. Two rounds of 2D classification and two rounds of 3D classification were performed to remove bad particles, followed by AutoRefine, Bayesian polish and contrast transfer function (CTF) refinement in Relion to improve the map quality. The final EM density map was generated by the non-uniform (NU) refinement in CryoSPARC. **b.** Local resolution distribution of the final map of the $Na_V1.6^{EM}$/β1/β2 complex. **c.** Angular distribution of the cryo-EM reconstruction of $Na_V1.6^{EM}$/β1/β2 complex used for final refinement. **d.** Fourier shell correlations (FSC) curves of the $Na_V1.6^{EM}$/β1/β2 complex.

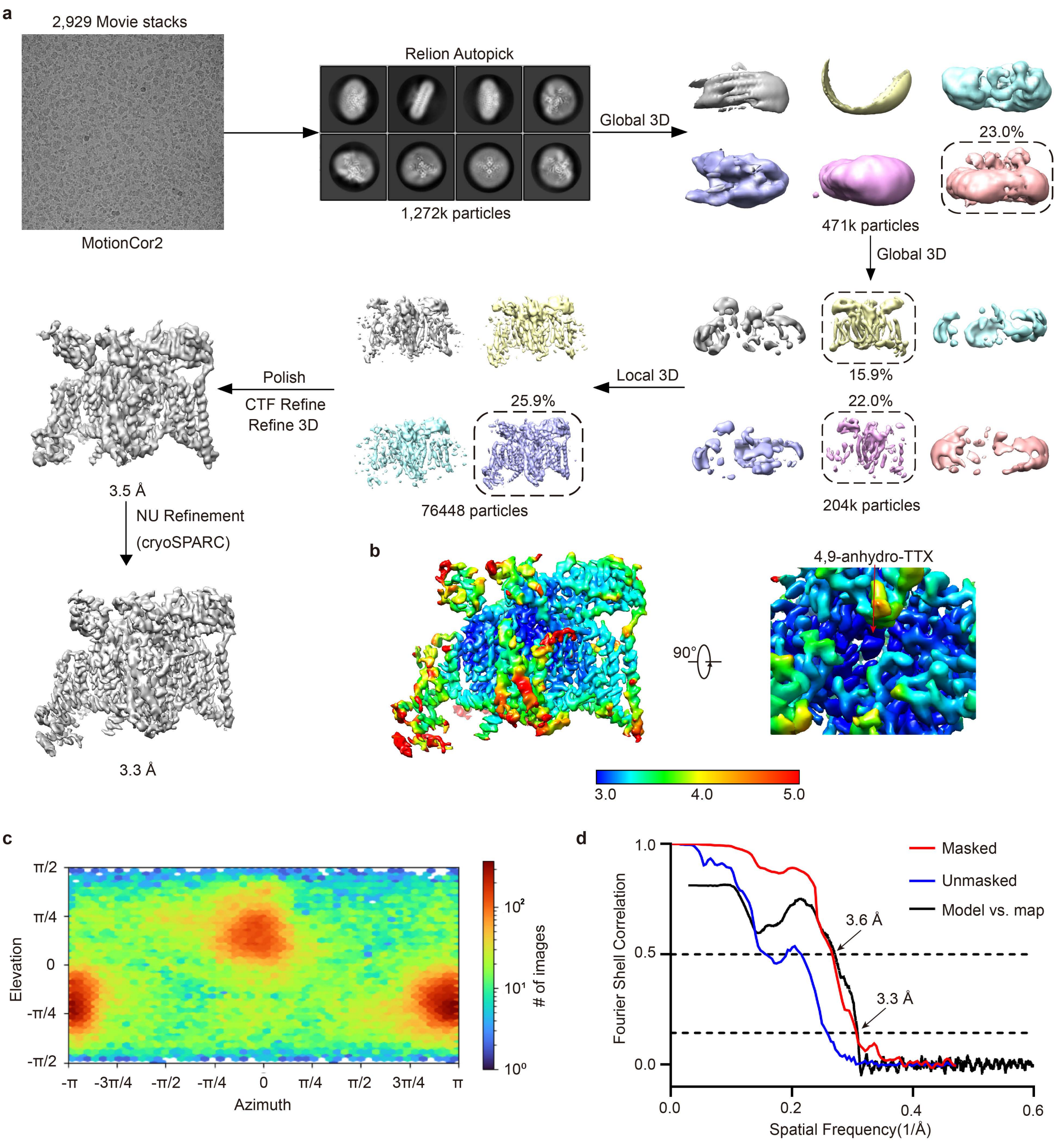

Supplementary Figure 4. Cryo-EM data processing of the $Na_V1.6^{4,9\text{-}ahTTX}$ complex

a. The flowchart of the $Na_V1.6^{EM}$/β1/β2 and 4,9-ah-TTX complex data processing. A total of 1,272k particles were picked from 2,929 micrographs. Two rounds of global 3D classification and a further round of local 3D classification were performed to remove bad particles, followed by AutoRefine, Bayesian polish and contrast transfer function (CTF) refinement in Relion to improve the map quality. The final EM density map was genera ted by the non-uniform (NU) refinement in CryoSPARC. **b.** Local resolution distribution of the final 3D map. The EM density of 4,9-anhydro-TTX was labelled with a red arrow. **c.** Angular distribution of the particles used for the final 3D reconstruction. **d.** Fourier shell correlations (FSC) curves of the $Na_V1.6^{4,9\text{-}ahTTX}$ complex.

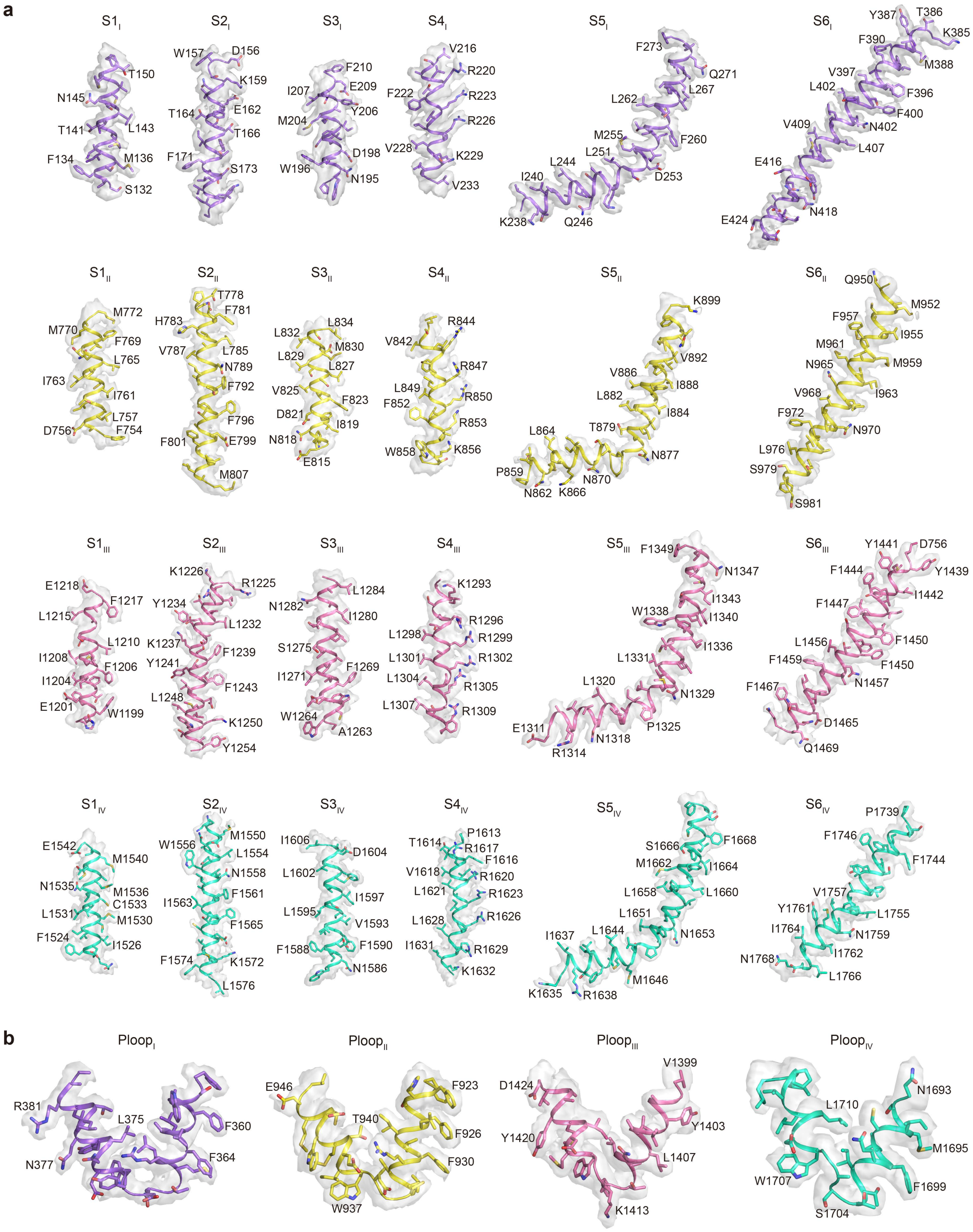

Supplementary Figure 5. Representative EM densities for $Na_V1.6^{EM}$/β1/β2 complex.

a. The EM densities for the S1–S6 segments in each domain were shown as gray surface. **b.** The EM densities for the Pore loop segments in each domain were shown as gray surface. Side-chains of selected residues were shown as sticks.

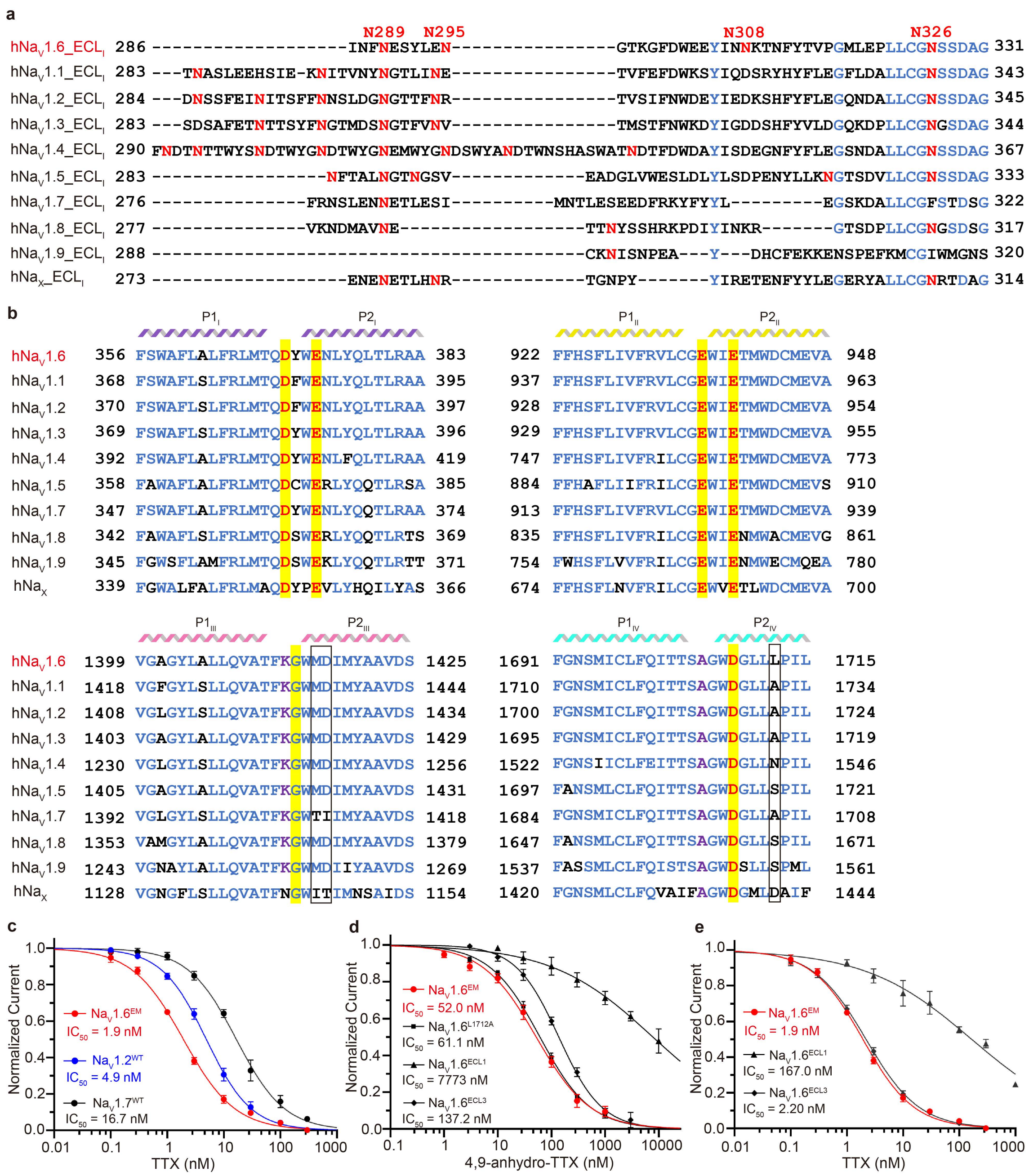

Supplementary Figure 6. Sequence alignments of the human Na_V channels and functional characterizations of human Na_V channels by TTX analogs.

a. Sequence alignment of the ECL_I between human Na_V channel subtypes. The identical residues were marked in blue. Asn linked glycosylation sites were marked in red and labelled. **b**. Sequence alignment of P1 and P2 of human Na_V channels. The main residues involved in 4,9-ah-TTX binding are highlighted in yellow. Positively charged residues and Lys/Ala were marked in red and purple, respectively. **c**. The concentration-response curves for the blockade of $Na_V1.6^{EM}$ (red), $Na_V1.2^{WT}$ (blue), and $Na_V1.7^{WT}$ (black) by TTX. $Na_V1.6^{EM}$, *n* = 5; $Na_V1.2^{WT}$, *n* = 5; $Na_V1.7^{WT}$, *n* = 4. **d**. The concentration-response curves for the blockade of $Na_V1.6^{EM}$ and its variants by 4,9-ah-TTX. $Na_V1.6^{EM}$, *n* = 5; $Na_V1.6^{L1712A}$, *n* = 4; $Na_V1.6^{ECL1}$, *n* = 5; $Na_V1.6^{ECL3}$, *n* = 4. **e**. The concentration-response curves for the blockade of $Na_V1.6^{EM}$ and its variants by TTX. $Na_V1.6^{EM}$, *n* = 5; $Na_V1.6^{ECL1}$, *n* = 7; $Na_V1.6^{ECL3}$, *n* = 4. Data are presented as mean ± SEM. *n* biological independent cells. Source data are provided as a Source Data file.

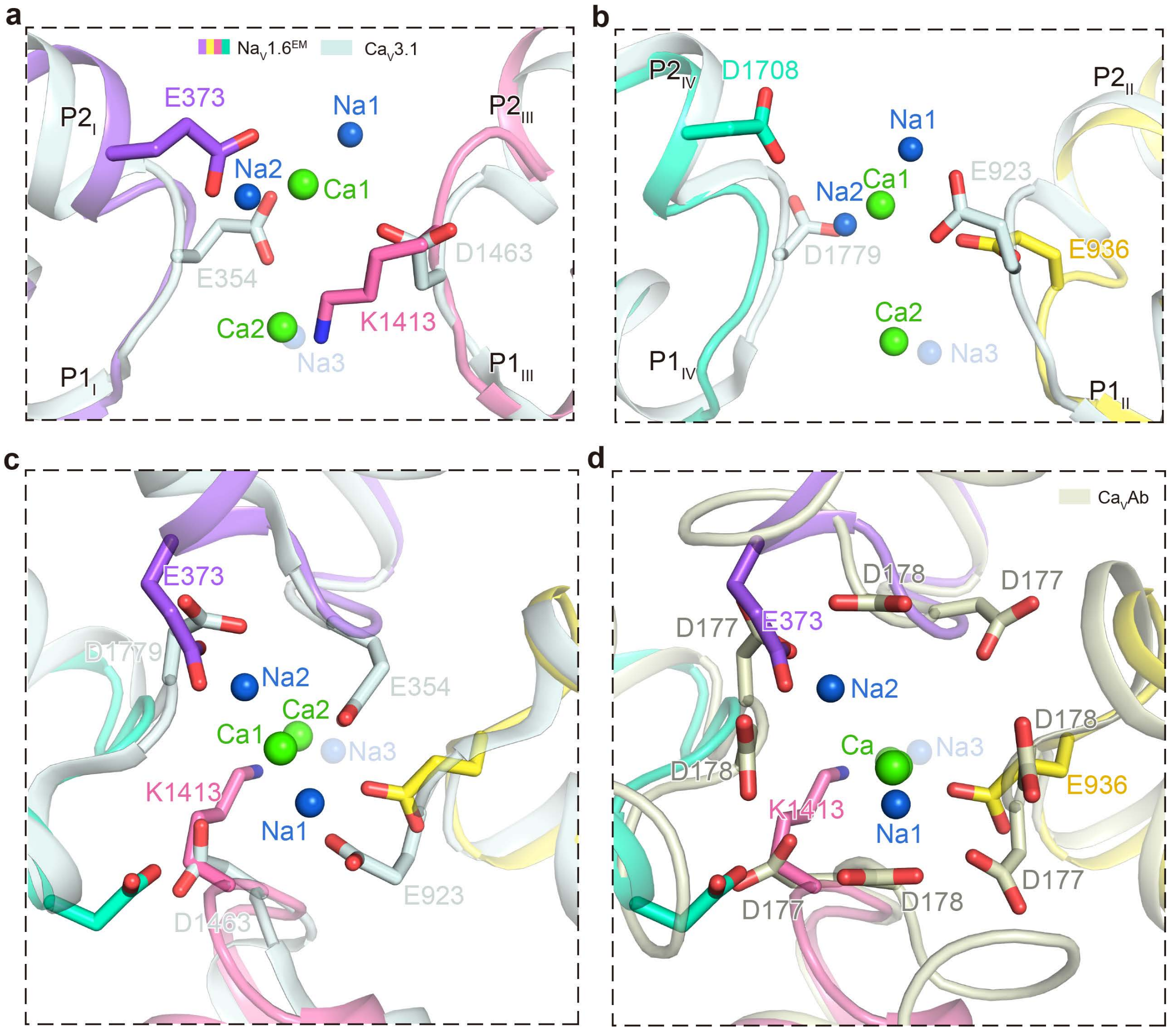

Supplementary Figure 7. Structural comparison of Na^+ binding sites of $Na_V1.6^{EM}$ and Ca^{2+} binding sites of $Ca_V3.1$.

a-b. Side view of the Na^+ sites compared with the Ca^{2+} sites of $Ca_V3.1$ (PDB code: 6KZO, colored in light blue). The diagonal repeats of DI and DIII (**a**), DII and DIV (**b**) are shown separately for clarity. **c-d.** Top-down view of the Na^+ sites of $Na_V1.6$ compared with the Ca^{2+} sites of $Ca_V3.1$ (PDB code: 6KZO, colored in light blue) (c) and that of Ca_VAb (PDB code: 4MS2, colored in gray) (d), respectively. Key residues were shown side-chains in sticks. Ions were shown as spheres.

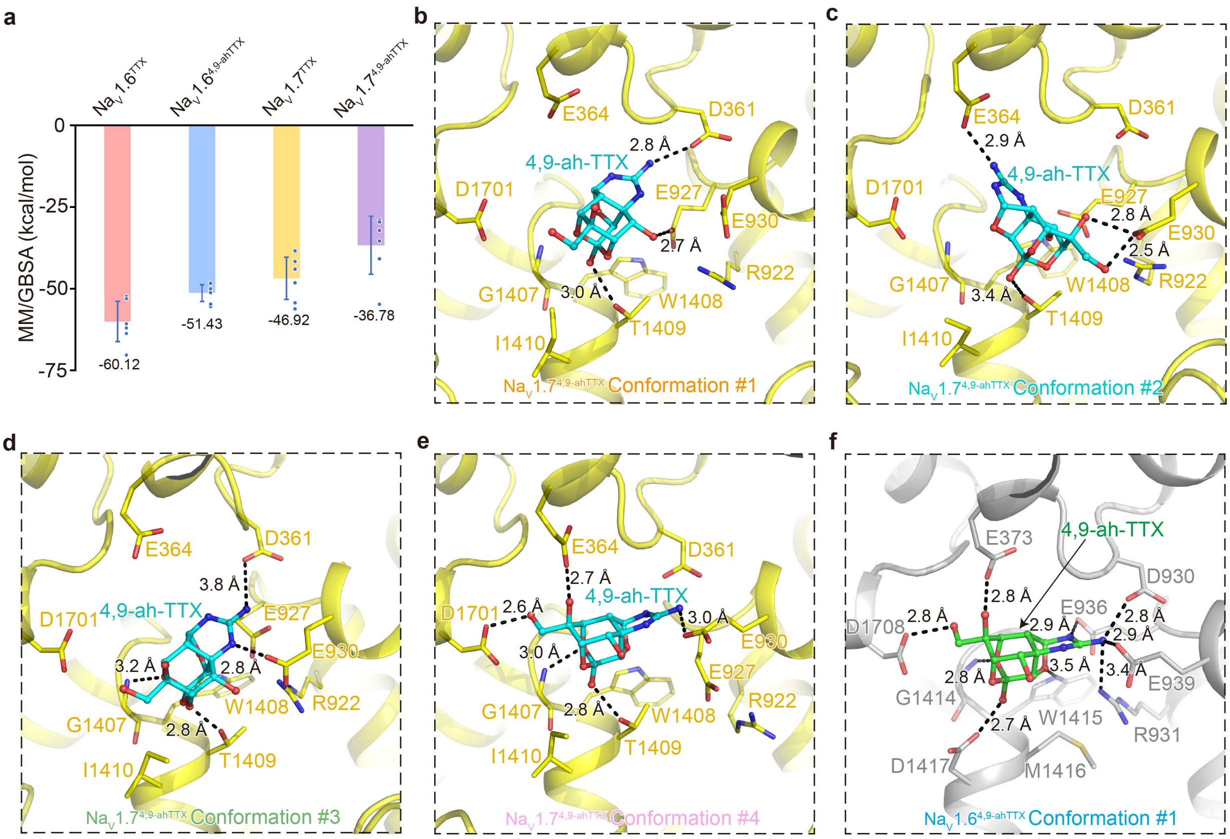

Supplementary Figure 8. Different conformations of 4,9-ah-TTX binding to $Na_V1.6$ and $Na_V1.7$ from MD.

a. The binding affinity of TTX and 4,9-ah-TTX to $Na_V1.6$ and $Na_V1.7$ calculated by MM/GBSA from the MD simulations. MD simulations for $Na_V1.6^{TTX}$, $Na_V1.6^{4,9\text{-}ahTTX}$, $Na_V1.7^{TTX}$, and $Na_V1.7^{4,9\text{-}ahTTX}$ were repeated 6 times independently. Data are presented as mean ± SEM. **b-e**. Detailed binding modes of the four major conformations of 4,9-ah-TTX binding in $Na_V1.7$ from the MD simulations. **f**. The only major binding mode of 4,9-ah-TTX in $Na_V1.6$ from the MD simulations. Source data are provided as a Source Data file.

Residues interacting with ligands	Na$_v$1.6$^{4,9\text{-ahTTX}}$		Na$_v$1.7$^{4,9\text{-ahTTX}}$					Na$_v$1.6^{TTX}				Na$_v$1.7^{TTX}			
	c.1	c.2	c.1	c.2	c.3	c.4	c.5	c.1	c.2	c.3	c.4	c.1	c.2	c.3	c.4
Cluster population (ns)	599	2	342	105	82	67	5	555	32	11	2	479	87	30	3
IP [GLU936 (GLU927)]	100%	100%	87%	97%	100%	28%	60%	100%	84%	100%	100%	100%	100%	100%	100%
HA [GLU936 (GLU927)]	100%	100%	71%	98%	80%	22%	80%	92%	9%	91%	100%	100%	100%	100%	100%
HA [GLU373 (GLU364)]	100%	100%	55%	48%	7%	97%	60%	100%	100%	82%	50%	100%	100%	100%	100%
IP [GLU939 (GLU930)]	98%	100%	21%		87%	78%		91%	34%	73%	100%	39%	76%	43%	33%
IP [ASP370 (ASP361)]	97%	100%	53%	1%	91%	100%	20%	100%	100%	100%	100%	100%	100%	100%	100%
HA [ASP370 (ASP361)]	96%	100%	52%	1%	80%	100%		100%	100%	100%	100%	98%	100%	100%	100%
HA [GLU939 (GLU930)]	93%	50%	46%	91%	91%	78%	100%	92%	31%	82%	100%	62%	70%	73%	67%
HD [MET1416 (THR1409)]	92%	100%	92%	54%	100%	97%	80%	99%	100%	100%	100%	100%	100%	100%	100%
HD [ASP1417 (ILE1410)]	79%	100%	30%		60%	60%		25%	100%	18%		65%	18%	70%	100%
HD [GLY1414 (GLY1407)]	75%	100%	92%	5%	98%	100%	20%	41%	100%	55%	100%	100%	100%	100%	100%
HA [ASP1417 (ILE1410)]	73%	50%						20%	100%	9%					
HD [TRP1415 (TRP1408)]	65%	50%	45%	10%	24%	97%	60%	46%	100%	45%		84%	85%	70%	67%
AR [TYR371 (TYR362)]	54%		21%	53%		61%	60%	32%	6%	9%		8%	15%		
HA [ASP1708 (ASP1701)]	51%	100%	37%		9%	99%		52%	100%			37%	54%	30%	
HD [ARG931 (ARG922)]	47%	50%	48%	65%	45%		80%	10%		9%		53%	2%	87%	100%
HY [TYR371 (TYR362)]	39%	50%	58%	82%		30%	60%	37%	9%	18%		23%	24%	7%	
HD [GLY1706 (GLY1699)]	12%		1%					36%	22%	18%		71%	84%	80%	100%
HA [GLY1414 (GLY1407)]	11%	50%	0%					19%	3%	18%					
HA [PHE1412 (PHE1405)]	5%		16%	27%				4%	31%	9%		11%	11%	20%	
HD [GLY1709 (GLY1702)]	5%	50%						23%		9%					
IP [GLU373 (GLU364)]	5%		38%	54%	20%		60%	34%		9%		73%	2%	100%	100%
HY [LYS1413 (LYS1406)]	1%														
HA [GLY1706 (GLY1699)]	1%							5%							
HA [TYR371 (TYR362)]	1%		7%				40%	1%			50%	2%	1%		
HD [TYR371 (TYR362)]	0%		3%					65%	100%	91%	100%	21%	91%	3%	
HD [ASN374 (ASN365)]	0%							4%							
HD [TRP1707 (TRP1700)]	0%														
HA [GLY317 (GLY308)]			1%												
HD [LEU319 (LYS310)]			2%	6%								0%			
HA [GLN369 (GLN360)]			5%			7%	20%	1%	16%			1%	9%		
HA [CYS934 (CYS925)]			1%			1%									
HA [GLY935 (GLY926)]			47%			93%						1%	16%		
HD [LYS1413 (LYS1406)]			1%					10%	100%			19%	57%		
AR [TRP1415 (TRP1408)]			1%	3%											
HA [MET1416 (THR1409)]			78%	6%	100%	97%		0%				99%	100%	100%	100%
IP [ASP1708 (ASP1701)]			13%	1%								0%			
HA [THR314 (TYR305)]				1%											
HD [TRP917 (TRP908)]				4%			20%								
HD [TYR1420 (TYR1413)]				21%			60%								
HD [TRP372 (TRP363)]								5%		9%					
HA [LYS1413 (LYS1406)]								20%		27%					

Supplementary Figure 9. Ligand-protein contact analysis based on MD study.

The type and frequency of interactions between protein and ligand are listed for each conformation cluster of a protein-ligand system. C.# indicates the index of the cluster. The digits associated with green-white color scheme indicate the appearance frequency of the interaction within the cluster. For residues interacting with ligands, the annotation follows the format of "Interaction Type [Residue in Na$_V$1.6 (Counterpart the residue in Na$_V$1.7)]". The interaction identification and interaction type definition follow the study described by Daria et al[1]. A general annotation for interaction types is listed below: IP (salt bridges), HY (hydrophobic interactions), HA (hydrogen bond, ligand atom as acceptor), HD (hydrogen bond, ligand atom as donor), AR (aromatic system related stacking).

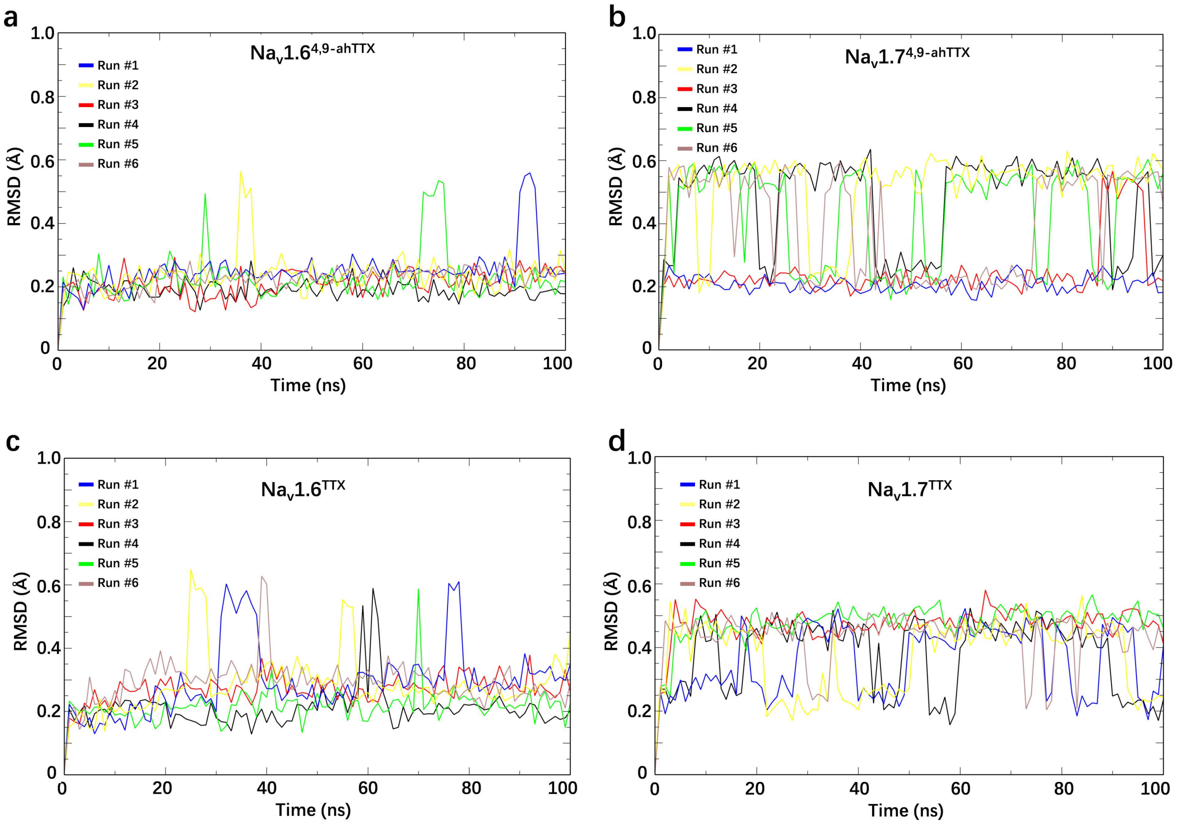

Supplementary Figure 10. Ligand dynamics in MD study.

a to **d**, The ligand RMSD plots for each replicate of the simulations for $Na_v1.6^{4,9\text{-}ahTTX}$ (a), $Na_v1.7^{4,9\text{-}ahTTX}$ (b), $Na_v1.6^{TTX}$ (c), and $Na_v1.7^{TTX}$ (d). Before RMSD calculation, all the structures in each trajectory were aligned with the initial structure of that trajectory by using Least Squares algorithm.

Supplementary Table 1. Concentration-response inhibition statistics of 4,9-ah-TTX or TTX for different subtypes and variants.

Human subtypes	Compounds	IC50 (nM)	95% CI	n
$Na_V1.6^{EM}$	4,9-ah-TTX	52.0	44.33 to 61.09	5
$Na_V1.2^{WT}$	4,9-ah-TTX	257.9	217.4 to 305.9	6
$Na_V1.7^{WT}$	4,9-ah-TTX	1340	1209 to 1486	6
$Na_V1.6^{ELC1}$	4,9-ah-TTX	7773	4538 to 13313	5
$Na_V1.6^{ELC3}$	4,9-ah-TTX	137.2	123.1 to 152.9	4
$Na_V1.6^{L1712A}$	4,9-ah-TTX	61.1	55.45 to 67.23	4
$Na_V1.6^{M1416T/D1417I}$	4,9-ah-TTX	256.5	224.0 to 293.7	5
$Na_V1.6^{EM}$	TTX	1.9	1.76 to 2.13	5
$Na_V1.2^{WT}$	TTX	4.9	4.43 to 5.46	5
$Na_V1.7^{WT}$	TTX	16.7	14.54 to 19.18	4
$Na_V1.6^{ELC1}$	TTX	167.0	115.2 to 242.3	7
$Na_V1.6^{ELC3}$	TTX	2.2	2.01 to 2.41	4

Supplementary Table 2. Primers used in this study.

Name	Sequence (5' to 3')
$Na_V1.6$ F	atggcagcgcggctgcttg
$Na_V1.6$ R	acacttggattctctgacctctttttgt
$Na_V1.6^{\triangle DI\text{-}DII}$ F	tcccctcggagctctcggaaggacagaatcaacagtata
$Na_V1.6^{\triangle DI\text{-}DII}$ R	gattctgtccttccgagagctccgaggggagccccctcc
$Na_V1.6^{\triangle DII\text{-}DIII}$ F	acagaggatgttagcggcaagtcttggtggatcctgcgg
$Na_V1.6^{\triangle DII\text{-}DIII}$ R	ccaccaagacttgccgctaacatcctctgtgttgaggtt
$Na_V1.6^{\triangle CTer}$ F	caccgggagaaaaaagagctggaagttctgttccaggggcccatggtgagcaa
$Na_V1.6^{\triangle CTer}$ R	ctggaacagaacttccagctcttttttctcccggtgtgtgcctccattctccagc
$Na_V1.6^{L1712A}$ F	gatggcctgctgGCGcccatcctaaaccgcccccctgact
$Na_V1.6^{L1712A}$ R	ggtttaggatgggCGCcagcaggccatcccaaccagctga
$Na_V1.6^{ECL1}$ F	ctgcagctgttcatggggaaccttaagcataaatgttttcgaaattca
$Na_V1.6^{ECL1}$ R	agtgtcaaaacttgtgtaaccataatcagggtttctgccaattttcac
$Na_V1.6^{ECL3}$ F	ttcagcatcatgggagttaacttgtttgctggcaagttctatgagtgt
$Na_V1.6^{ECL3}$ R	aagaagggccaggtatcctgccccgacattatcaaagttcactttcag
$Na_V1.6^{M1416T/D1417I}$ F	ccttcaaaggctggacgattatcatgtatgcagctgtagattcccggaagcctg
$Na_V1.6^{M1416T/D1417I}$ R	cagctgcatacatgataatcgtccagcctttgaaggttgctacttgaagaagggc

Supplementary Table 3. Cryo-EM data collection, refinement and validation statistics

	$Na_V1.6^{EM}/\beta1/\beta2$ (EMDB-34387) (PDB: 8GZ1)	$Na_V1.6^{4,9\text{-}ahTTX}$ (EMDB-34388) (PDB: 8GZ2)
**Data collection and processing**		
Magnification	130,000 ×	130,000 ×
Voltage (kV)	300	300
Electron exposure (e–/Å^2)	60	60
Defocus range (μm)	−1.2 ~ −2.2	−1.2 ~ −2.2
Pixel size (Å)	1.04	1.04
Symmetry imposed	C1	C1
Initial particle images (no.)	1594472	1272486
Final particle images (no.)	41387	76448
Map resolution (Å)	3.4	3.3
FSC threshold	0.143	0.143
Map resolution range (Å)	3.0 ~ 5.0	3.0 ~ 5.0
**Refinement**		
Initial model used (PDB code)	6J8I	Na_V1.6-β1-β2
Model resolution (Å)	3.7	3.6
FSC threshold	0.5	0.5
Map sharpening *B* factor (Å^2)	-69.0	-58.1
Model composition		
Non-hydrogen atoms	11912	11984
Protein residues	1445	1467
Ligands	19	18
B factors (Å^2)		
Protein	90.46	79.36
Ligand	98.52	75.52
R.m.s. deviations		
Bond lengths (Å)	0.003	0.005
Bond angles (°)	0.623	0.818
Validation		
MolProbity score	1.89	2.03
Clashscore	10.55	12.37
Poor rotamers (%)	0.00	0.00
Ramachandran plot		
Favored (%)	94.98	93.62
Allowed (%)	5.02	6.38
Disallowed (%)	0.00	0.00

SUPPLEMENTARY REFERENCES

1 Kokh, D. B. *et al.* A workflow for exploring ligand dissociation from a macromolecule: Efficient random acceleration molecular dynamics simulation and interaction fingerprint analysis of ligand trajectories. *J Chem Phys* **153** (2020).
