# Supplementary material for: Structure of human NaV1.6 channel reveals Na+ selectivity and pore blockade by 4,9-anhydro-tetrodotoxin
Source: Nat Commun. 2023 Feb 23;14:1030. doi: 10.1038/s41467-023-36766-9 (PMC9950489; doi:10.1038/s41467-023-36766-9)
Supplement: Supplementary file 3 — Reporting Summary [file 41467_2023_36766_MOESM3_ESM.pdf]

nature portfolio

Corresponding author(s): Yan Zhao, Zhuo Huang & Daohua Jiang

Last updated by author(s): Feb 2, 2023

Reporting Summary

Nature Portfolio wishes to improve the reproducibility of the work that we publish. This form provides structure for consistency and transparency in reporting. For further information on Nature Portfolio policies, see our Editorial Policies and the Editorial Policy Checklist.

Statistics

For all statistical analyses, confirm that the following items are present in the figure legend, table legend, main text, or Methods section.

n/a	Confirmed	
☐	☒	The exact sample size (*n*) for each experimental group/condition, given as a discrete number and unit of measurement
☐	☒	A statement on whether measurements were taken from distinct samples or whether the same sample was measured repeatedly
☐	☒	The statistical test(s) used AND whether they are one- or two-sided<br>*Only common tests should be described solely by name; describe more complex techniques in the Methods section.*
☒	☐	A description of all covariates tested
☐	☒	A description of any assumptions or corrections, such as tests of normality and adjustment for multiple comparisons
☐	☒	A full description of the statistical parameters including central tendency (e.g. means) or other basic estimates (e.g. regression coefficient) AND variation (e.g. standard deviation) or associated estimates of uncertainty (e.g. confidence intervals)
☐	☒	For null hypothesis testing, the test statistic (e.g. *F*, *t*, *r*) with confidence intervals, effect sizes, degrees of freedom and *P* value noted<br>*Give P values as exact values whenever suitable.*
☒	☐	For Bayesian analysis, information on the choice of priors and Markov chain Monte Carlo settings
☒	☐	For hierarchical and complex designs, identification of the appropriate level for tests and full reporting of outcomes
☒	☐	Estimates of effect sizes (e.g. Cohen's *d*, Pearson's *r*), indicating how they were calculated

Our web collection on statistics for biologists contains articles on many of the points above.

Software and code

Policy information about availability of computer code

Data collection	All cryo-EM data were collected using SerialEM v3.8.4, electrophysiological data were collected using Patchmaster2019.
Data analysis	Collected cryo-EM data were processed using Relion 3, MotionCorr 2, Gctf, cryoSPARC v3.2.0. Map and model refinement were processed with UCSF-Chimera v1.14, COOT v0.8.9.2, Phenix v1.17.1. Electrophysiology data were acquired using PatchMaster2019 and processed using Origin 2020b. Molecular Dynamics simulations were performed and analyzed using GAFF2, LINCS, Parrinello–Rahman, and GROMACS 2021. All figures were analyzed and made using Pymol v2.4.0, ChimeraX v1.1, Excel2016, Jalview1.8.3 and Graphpad v9.1.1.

For manuscripts utilizing custom algorithms or software that are central to the research but not yet described in published literature, software must be made available to editors and reviewers. We strongly encourage code deposition in a community repository (e.g. GitHub). See the Nature Portfolio guidelines for submitting code & software for further information.

Data

Policy information about availability of data

All manuscripts must include a data availability statement. This statement should provide the following information, where applicable:

- Accession codes, unique identifiers, or web links for publicly available datasets
- A description of any restrictions on data availability
- For clinical datasets or third party data, please ensure that the statement adheres to our policy

The UniProt accession codes for the sequences of human Nav1.6, β1 and β2 are Q9UQD0 [https://www.uniprot.org/uniprot/Q9UQD0], Q07699 [https://

www.uniprot.org/uniprot/Q07699], and O60939 [https://www.uniprot.org/uniprot/O60939], respectively. The accession codes for the coordinates of Nav1.7, CaVAb, and CaV3.1 used in this study are 6J8J [http://doi.org/10.2210/pdb6J8J/pdb], 4MS2 [http://doi.org/10.2210/pdb4MS2/pdb], and 6KZO [http://doi.org/10.2210/pdb6KZO/pdb], respectively. The three-dimensional cryo-EM density maps of the human NaV1.6/β1/β2 and NaV1.6/β1/β2-4,9-anhydro-TTX have been deposited in the EM Database under accession codes EMD-34387 [https://www.emdataresource.org/EMD-34387] and EMD-34388 [https://www.emdataresource.org/EMD-34388], respectively. The coordinates of the NaV1.6/β1/β2 and NaV1.6/β1/β2-4,9-anhydro-TTX have been deposited in the Protein Data Bank under accession codes 8GZ1 [http://doi.org/10.2210/pdb8GZ1/pdb] and 8GZ2 [http://doi.org/10.2210/pdb8GZ2/pdb], respectively.

Human research participants

Policy information about studies involving human research participants and Sex and Gender in Research.

Reporting on sex and gender	N/A
Population characteristics	N/A
Recruitment	N/A
Ethics oversight	N/A

Note that full information on the approval of the study protocol must also be provided in the manuscript.

Field-specific reporting

Please select the one below that is the best fit for your research. If you are not sure, read the appropriate sections before making your selection.

☒ Life sciences ☐ Behavioural & social sciences ☐ Ecological, evolutionary & environmental sciences

For a reference copy of the document with all sections, see nature.com/documents/nr-reporting-summary-flat.pdf

Life sciences study design

All studies must disclose on these points even when the disclosure is negative.

Sample size	Sample sizes were not predetermined for this study. For the cryo-EM studies, the number of micrographs is determined by the available microscope time. The reconstruction map resolution was sufficient to build accurate protein 3D model. For the electrophysiology experiments, recording results from 3 different cells are usually acceptable. The authors have recorded 6 or more cells/patches for each individual functional array. Increasing the sample size, does not change our results. And data for each assay were acquired from at least three independently transfected cells. The sample sizes of all these experiments were determined based on the consistency and variability.
Data exclusions	For cryo-EM analysis, micrographs with CTF fitting worse than 4 angstrom were discarded. Following cryo-EMdata processing only kept high-resolution and homogeneous particles to generate the final high resolution map.
Replication	Sample preparation related experiments including purification and SDS-PAGE gel electrophoresis were reproduced at least three times independently. Whole cell patch clamp recording and MD simulations were also reproduced at least three times independently. All attempts at replication were successful.
Randomization	All particles were aligned to a resolution low-passed initial model, and divided randomly into several classes. As the translation and rotation operations went precisely, homogeneous particles went to the same class. For electrophysiology experiments, randomization is not relevant as no group allocations were performed.
Blinding	Not applicable for this study. For the cryo-EM studies, all the data were collected automatically. For electrophysiology experiments, cells selected for data measurement were indicated based on a GFP fluorescent protein signal.

Reporting for specific materials, systems and methods

We require information from authors about some types of materials, experimental systems and methods used in many studies. Here, indicate whether each material, system or method listed is relevant to your study. If you are not sure if a list item applies to your research, read the appropriate section before selecting a response.

Materials & experimental systems

n/a	Involved in the study
☒	☐ Antibodies
☐	☒ Eukaryotic cell lines
☒	☐ Palaeontology and archaeology
☒	☐ Animals and other organisms
☒	☐ Clinical data
☒	☐ Dual use research of concern

Methods

n/a	Involved in the study
☒	☐ ChIP-seq
☒	☐ Flow cytometry
☒	☐ MRI-based neuroimaging

Eukaryotic cell lines

Policy information about cell lines and Sex and Gender in Research Sf9 cells, Invitrogen, 11496015; HEK293F, Gibco, 11625019.

Cell line source(s)	Sf9(Invitrogen, USA), HEK293-F (FreeStyle 293-F cells, Gibco, USA); HEK293T (Gibco, USA)
Authentication	We did not anthenticate theSF9, HEK293T or HEK293-F cell line.
Mycoplasma contamination	The cell lines were not tested for mycoplasma contamination.
Commonly misidentified lines (See ICLAC register)	No other cell line used in this study.
