# Supplementary figures and images for: Structure of human NaV1.6 channel reveals Na+ selectivity and pore blockade by 4,9-anhydro-tetrodotoxin
Source: Nat Commun. 2023 Feb 23;14:1030. doi: 10.1038/s41467-023-36766-9 (PMC9950489; doi:10.1038/s41467-023-36766-9)

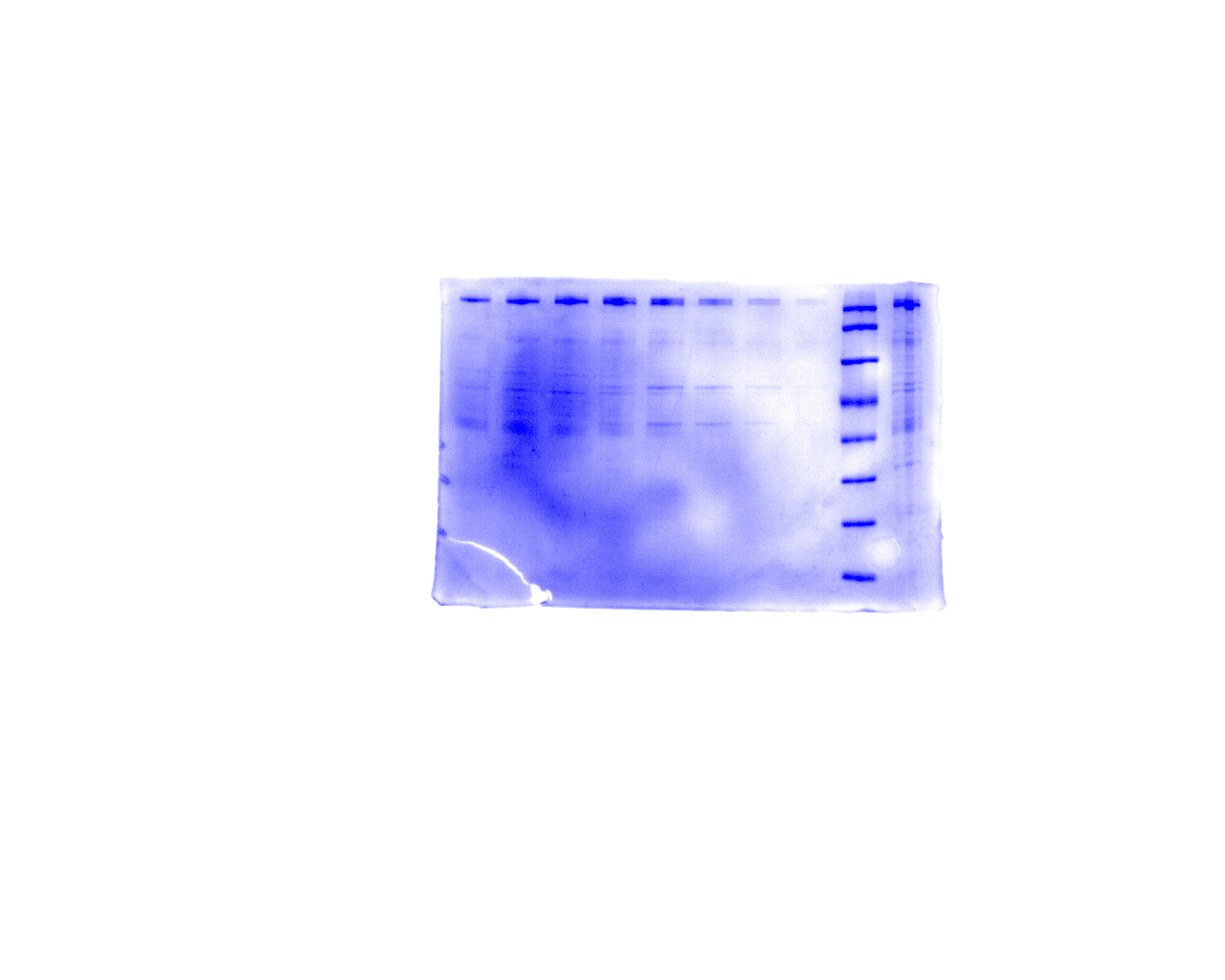

Supplement: Supplementary file 4 — Source Data [file 41467_2023_36766_MOESM4_ESM.zip › Source Data/Source_Data_1-Supplementary_Figure_2b.BMP]
